# Complete Genome Sequence of Fish Pathogen *Aeromonas hydrophila* AL06-06

**DOI:** 10.1128/genomeA.00368-15

**Published:** 2015-04-23

**Authors:** Hasan C. Tekedar, Attila Karsi, Ali Akgul, Safak Kalindamar, Geoffrey C. Waldbieser, Tad Sonstegard, Steven G. Schroeder, Mark L. Lawrence

**Affiliations:** a College of Veterinary Medicine, Mississippi State University, Mississippi State, Mississippi, USA; b Warmwater Aquaculture Research Unit, Agriculture Research Service, United States Department of Agriculture, Stoneville, Mississippi, USA; c Bovine Functional Genomics Laboratory, Agricultural Research Service, United States Department of Agriculture, Beltsville, Maryland, USA

## Abstract

*Aeromonas hydrophila* occurs in freshwater environments and infects fish and mammals. Here, we report the complete genome sequence of *Aeromonas hydrophila* AL06-06, which was isolated from diseased goldfish and is being used for comparative genomic studies with *A. hydrophila* strains that cause bacterial septicemia in channel catfish aquaculture.

## GENOME ANNOUNCEMENT

*Aeromonas* species are Gram-negative facultative anaerobes that have worldwide distribution in aquatic environments (1, 2), and they can be isolated from domesticated pets, invertebrate species, birds, ticks, insects, and natural soils (3). Also, many aeromonads cause disease in fish. *Aeromonas hydrophila* is a motile species that has primarily been considered an opportunistic pathogen in fish and humans (3). We previously reported the complete genome sequence of *A. hydrophila* ML09-119 (1), which represents a clonal group of *A. hydrophila* strains causing outbreaks of bacterial septicemia in commercial channel catfish aquaculture in the southeastern United States. We now report the complete genome sequence of *A. hydrophila* AL06-06, which was isolated from a diseased goldfish in 2006 from the Auburn University Southeastern Cooperative Fish Disease Laboratory in Greensboro, Alabama. The AL06-06 genome sequence will be used for comparative genomic purposes with other sequenced *Aeromonas* strains, particularly those causing disease in catfish.

The genome of *A. hydrohila* AL06-06 was fully sequenced using an Illumina Genome Analyzer IIx (a total of 6,629,874 reads, with 150× coverage). Trimming, error correction, contig creation, and quality control of sequence reads were conducted using CLC Workbench 6.5.1 (CLC Bio) and Sequencher 5.2.3 (Gene Codes Corporation). *De novo* assembly was performed by CLC Workbench 6.5.1. Scaffolded gaps were closed by Sanger sequencing of PCR amplicons. For the unscaffolded gaps, single-primer PCR was used for amplification of sequence templates (4). Ribosomal operons and other repetitive regions were amplified and completely resequenced to create a reliable assembly.

NCBI’s Prokaryotic Genomes Automatic Annotation Pipeline (PGAAP) (5) was used for AL06-06 genome annotation, followed by submission to GenBank. The complete genome of *A. hydrophila* genome comprises 4,884,823 bp with 61.3% G+C content. It contains 4,453 predicted genes, of which 4,235 are protein coding. A total of 112 tRNAs and 10 rRNA operons were predicted by PGAAP.

The *A. hydrophila* AL06-06 genome was also annotated by RAST (6) to facilitate comparison with the *A. hydrophila* ML09-119 genome. Based on functional comparative results, *A. hydrophila* AL06-06 has 81 unique elements including arsenic resistance genes, heme and hemin uptake-utilization systems, some membrane transport genes for type I and type V secretion systems, transposable elements, and nitrogen metabolism genes. In particular, strain AL06-06 has a specific arsenic resistance mechanism that is missing in the *A. hydrophila* ML09-119 genome. Due to its ubiquitous distribution in the environment, *A. hydrophila* is prone to arsenic exposure (7). The *A. hydrophila* AL06-06 genome also has three plasmids compared to strain ML09-119, which does not carry any plasmids.

In summary, the complete genome of *A. hydrophila* AL06-06 contributes to our knowledge of *A. hydrophila* virulence and environmental adaptations, and it is especially useful for comparison with other fish- and human-pathogenic *A. hydrophila* strains.

### **Nucleotide sequence accession number**s. 

The completed genome sequence of *A. hydrophila* AL06-06 was deposited in GenBank under the accession no. CP010947 (the version described in this paper is CP010947.1, GI:764079125). The accession numbers for the plasmids are CP010948 (for pAH06-06-1; the version described in this paper is version CP010948.1, GI: 764083361), CP010949 (for pAH06-06-2; the version described in this paper is version CP010949.1, GI:764083365), and CP010950 (for pAH06-06-3; the version described in this paper is version CP010950.1, GI:764083373).
